# Increased Mobile Zinc Regulates Retinal Ganglion Cell Survival via Activating Mitochondrial OMA1 and Integrated Stress Response

**DOI:** 10.3390/antiox11102001

**Published:** 2022-10-10

**Authors:** Jiahui Tang, Zhe Liu, Jiaxu Han, Jingfei Xue, Liyan Liu, Jicheng Lin, Caiqing Wu, Qi Zhang, Siting Wu, Canying Liu, Haishun Huang, Yuanyuan Fu, Min Li, Yehong Zhuo, Yiqing Li

**Affiliations:** 1 State Key Laboratory of Ophthalmology, Zhongshan Ophthalmic Center, Sun Yat-sen University, Guangdong Provincial Key Laboratory of Ophthalmology and Visual Science, Guangzhou 510060, China; 2 Guangdong Provincial Key Laboratory of Chiral Molecule and Drug Discovery, School of Pharmaceutical Sciences, Sun Yat-Sen University, Guangzhou 510006, China

**Keywords:** optic nerve injury, retinal ganglion cells, zinc, mitochondria, ISR

## Abstract

Retinal ganglion cells (RGCs), the projection neurons of the eye, are irreversibly lost once the optic nerve is injured, which is a critical mechanism of glaucoma. Mobile zinc (Zn^2+^) levels rapidly increase in retinal interneuron amacrine cells and Zn^2+^ is then transferred to RGCs via the Zn^2+^ transporter protein ZnT-3, triggering RGC loss in optic nerve injury. Zn^2+^ chelation and ZnT-3 deletion promote long-term RGC survival. However, the downstream signaling pathways of Zn^2+^ in RGCs remains unknown. Here, we show that increased levels of Zn^2+^ upregulate the expression and activity of mitochondrial zinc metallopeptidase OMA1 in the retina, leading to the cleavage of DELE1 and activation of cytosolic eIF2α kinase PKR, triggering the integrated stress response (ISR) in RGCs. Our study identified OMA1 and ISR as the downstream molecular mechanisms of retinal Zn^2+^ and potential targets for preventing the progression of Zn^2+^-associated neuronal damage.

## 1. Introduction

**Figure 1:**
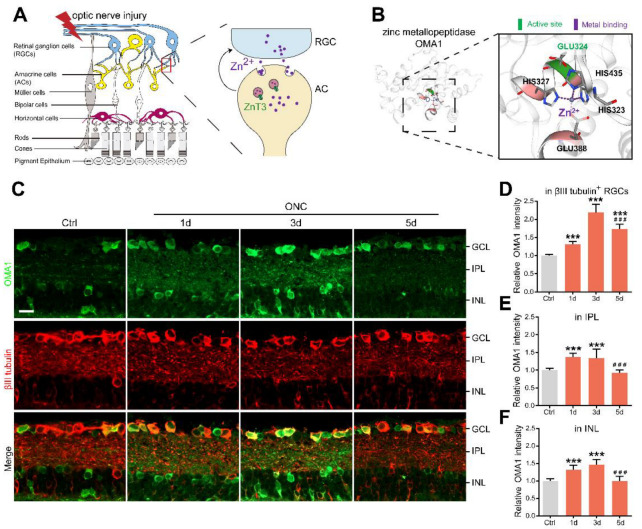
AC-to-RGC transfer of Zn^2+^ and OMA1 upregulation in optic nerve injury: (**A**). Schematic of retinal cross-sections (left) and the transsynaptic process of Zn^2+^ in the retina after optic nerve injury (right). (**B**). 3D models of mouse OMA1 based on the X-ray structure of the putative Zn^2+^-dependent peptidase Q74D82 at a resolution of 1.7 Å (left), showing the active and metal-binding sites (right). (**C**). OMA1 and βIII tubulin immunofluorescence staining in control and optic nerve injury murine retinas. Scale bar, 20 μm. (**D**–**F**). Quantitation of OMA1 expression in βIII tubulin-positive RGCs, IPL, and INL before and after ONC in wild-type mice (normalized to normal control; n = 7, 6, 6, 6). One-way ANOVA with Bonferroni post hoc tests, *** *p* < 0.001 compared with normal retina controls, ### *p* < 0.001 compared with 3 d post-ONC. GCL, ganglion cell layer; IPL, inner plexiform layer; INL, inner nuclear layer. All bars show the mean ± SEM.

Irreversible optic nerve injury in glaucoma progression presents an urgent clinical challenge [1]. A hallmark of glaucoma is progressive damage to the retinal ganglion cells (RGCs). Promoting RGC survival is a potential prerequisite for retina–brain connection regeneration and visual function recovery [2]. However, the mechanisms underlying the degeneration of RGCs in glaucomatous nerve injury remain unclear. Our previous studies have indicated that zinc dysregulation between retinal interneurons and projection neurons is a critical factor in RGC injury [3]. Mechanistically, mobile zinc (Zn^2+^) release from presynaptic interneuron amacrine cells (ACs) is increased within an hour after the optic nerve is injured, which is then transferred to postsynaptic projection neuron RGCs via synaptic vesicles, resulting in RGC injury (Figure 1A). The production of nitric oxide after optic nerve injury might act upstream of zinc liberation [4]. Elimination of Zn^2+^ by chelators or genetic modifications leads to both persistent RGC survival and substantial axon regeneration [3,5,6]. However, the downstream mechanisms of Zn^2+^ in postsynaptic RGC somatic injury have not been elucidated.

Mitochondria are involved in various biological processes, such as energy metabolism, signaling, reactive oxygen species (ROS) production, Ca^2+^ regulation, anti-pathogenic mechanisms, and programmed cell death (e.g., apoptosis and ferroptosis) [7,8,9,10]. Recent evidence has suggested mitochondrial vulnerability, such as NAD^+^ decline, mitochondrial fission increase, and mitochondrial unfolded protein response, as an emerging hypothesis for the pathogenesis of RGC injury [11,12]. Some mitochondria-targeted treatments (e.g., vitamin B3) have been shown to offer RGC protection in animal models and clinical trials [12,13]. Zn^2+^ participates in various mitochondrial biochemical reactions [14]. Under various stimuli, cytosolic zinc metalloproteins liberate zinc and increase the intracellular Zn^2+^ level. The cellular Zn^2+^ could: (1) form a cytosolic labile zinc pool and mediate ROS generation; (2) move into the mitochondria and other subcellular compartments (e.g., endoplasmic reticulum); and (3) be transported to the postsynaptic neurons by synaptic vesicles [11]. Notably, Zn^2+^ overload of mitochondria leads to mitochondrial depolarization, excessive fission, and increased permeability, resulting in neuronal death [15,16,17,18]. Thus, the relative stability of Zn^2+^ concentrations in the transsynaptic microenvironment is critical for neuronal mitochondrial homeostasis. However, the mechanisms by which Zn^2+^ mediates mitochondrial abnormalities remain unclear.

Overlapping with the m-AAA protease 1 homolog (OMA1) is a zinc metalloprotease located in the mitochondrial inner membrane (Figure 1B) [19,20]. Under various stress stimuli, OMA1 is activated to mediate the proteolytic processing of optic nerve atrophy protein (OPA1) at the S1 site, a protein that regulates the formation and maintenance of crista, mitochondrial fusion, and ATP production [21,22]. OMA1 activation and OPA1 overprocessing can inhibit mitochondrial fusion, promote cytochrome c release, and facilitate apoptosis [23,24,25]. Interestingly, OMA1 silencing has been reported to confer marked protection against a broad spectrum of experimental tissue damage, including to the brain and the cardiac muscle [26,27]. In addition to its canonical roles, activated OMA1 cooperates with death-associated protein 3 (DAP3) binding cell death enhancer 1 (DELE1) to relay mitochondrial malfunction to the cytosol and trigger the integrated stress response (ISR) [28,29]. DAP3, a subunit of mitoribosome, is involved in mitochondrial homeostasis and protein synthesis [30]. It also functions as a positive mediator of apoptosis [31]. DELE1 binds to DAP3 in mammalian cells. The cells stably expressing DELE are susceptible to apoptosis [32]. Additionally, DELE1 responds to compromised presequence processing by the mutated matrix proteases in neurodegenerative diseases [33]. ISR is an evolutionarily conserved stress-signaling network that regulates the initiation of cellular translation and reprograms gene expression according to organismal needs, by inducing phosphorylation of the eukaryotic translation initiation factor 2 α (eIF2α) in mammalian cells [34]. Chronic activation of ISR initiates a cell death program via increased production of proapoptotic components and has been associated with several neurological disorders [35,36,37]. Therefore, it is essential to elucidate the role of OMA1 and the mechanisms underlying its activation. However, unlike traditional ATP-dependent mitochondrial proteases (e.g., m-AAA), the activity of OMA1 is not affected by ATP absence, and its upstream regulatory mechanisms are not well understood.

Here, we report that the transsynaptic process (AC-to-RGC) of Zn^2+^ after optic nerve injury activates retinal OMA1 and leads to the cleavage of DELE1, which, together with eIF2α kinase 2 protein kinase R (PKR), stimulates phosphorylation of eIF2α and reprogramming of ISR-related gene expression. The elimination of Zn^2+^ suppressed OMA1, inhibited ISR in RGCs, and promoted RGC survival. Therefore, the Zn^2+^–OMA1–ISR axis represents a potential downstream mitochondrial pathway of increased Zn^2+^ in RGCs and the upstream regulatory mechanism for the activation of OMA1. These findings could assist in the development of future strategies for modulating the cellular response to Zn^2+^-induced mitochondrial dysfunction in human diseases.

## 2. Materials and Methods

### 2.1. Animals

C57BL/6J mice, ZnT-3 conditional knockout mice (Gempharmatech Co., Ltd., Nanjing, China), and littermate controls were used in this study. Mice were housed in standard cages in a specific pathogen-free facility on a 12 h light/dark cycle with ad libitum access to food and water. Mice were randomly allocated for each experiment. Regarding the sex factor, which is generally irrelevant to eye morphology [38], both male and female mice were randomly used. Optic nerve surgeries were performed on 6 to 8 weeks old mice (average body weight, 20–26 g) under general anesthesia, as previously described [39]. Immediately after optic nerve crush (ONC), single- or combined-use of TPEN (100 μM; 616394, Millipore, Burlington, MA, USA), C16 (4, 20, 100 μM; I9785, Sigma-Aldrich, St. Louis, MO, USA), ISRIB (10, 100, 1000 μM; SML0843, Sigma-Aldrich), or vehicle was administered by intraocular injection (3 μL per eye).

### 2.2. Standard Histological Procedures

Following perfusion and fixation of the heart, tissues to be used for immunostaining underwent brief postfixation (1 h at 4 °C) and dehydration with sucrose, before frozen sectioning at a thickness of 14 μm. After blocking with normal donkey serum (ASL050, Acmec biochemical, Shanghai, China), sections were incubated overnight at 4 °C with primary antibodies. Sections were washed 3 times, incubated with the appropriate fluorescent secondary antibodies (1:500; Abcam (Cambridge, UK) and Thermo Fisher Scientific (Waltham, MA, USA)) for 2 h at 25 °C, washed thrice, stained with 4′, 6-diamidino-2-phenylindole (DAPI, C02-04002, Bioss, Woburn, MA, USA), and mounted with antifading mounting medium (S2100, Solarbio, Beijing, China) and microscope cover glass (10212450C, CITOTEST, Rubano, Italy). Images were taken using a Zeiss LSM980 confocal microscope and a Nikon Eclipse Ni-U microscope.

### 2.3. Immunofluorescence Assay

Briefly, frozen-sectioned were incubated with primary antibodies at 4 °C overnight after blocking with appropriate sera for 1 h at RT. After washing three times, sections were incubated with the appropriate fluorescent secondary antibody and DAPI and then mounted. Primary antibodies that were used included: rabbit anti-OMA1 monoclonal (PA5-106421, 1:50; Thermo Fisher Scientific), mouse anti-βIII tubulin monoclonal (801202, 1:500, BioLegend, San Diego, CA, USA), mouse anti-AP2α monoclonal (3B5, 1:50; Developmental Studies Hybridoma Bank, Iowa City, IA, USA), rabbit anti-p-eIF2α monoclonal (D9G8, 1:50, Cell Signaling, Danvers, MA, USA), rabbit anti-ATF4 (monoclonal, D4B8, 1:50, Cell Signaling; polyclonal, 10835-1-AP, 1:50, Proteintech, Rosemont, IL, USA), mouse anti-ATF4 monoclonal (SC-390063, 1:50, Santa Cruz Biotechnology, Dallas, TX, USA), guinea pig anti-parvalbumin polyclonal (195004, 1:500, Synaptic System, Göttingen, Germany), mouse anti-CRALBP monoclonal (ab15051, 1:500, Abcam), mouse anti-PKCα monoclonal (SC-80, 1:200, Santa Cruz Biotechnology), and rabbit anti-TOMM20 polyclonal (11802-1-AP, 1:50, Proteintech). Fluorescence signals were quantified using ImageJ.

### 2.4. Quantitation of RGC Survival

Mice were administered an overdose of anesthesia 14 d after ONC. Perfusion and fixation were performed using isotonic saline and 4% (*w*/*v*) paraformaldehyde (PFA). Whole eyes were dissected and postfixed in 4% PFA for 1 h. RGC survival was evaluated by immunostaining using an antibody against βIII-tubulin followed by incubation with an Alexa Fluor 488-conjugated secondary antibody to rabbit IgG. Retinas were dissected and stained as whole-mounts after making 4 cuts to enable tissue to be flattened for photography. The ImageJ software was used to count βIII tubulin-positive cells using 4 images per retina (200×, Eclipse Ni-U microscope, Nikon, Tokyo, Japan), which were then averaged to estimate overall RGC survival.

### 2.5. Western Blot Analysis

Briefly, total retinal protein was extracted for Western blot assays. The PVDF membranes (IPVH00010, Merck Millipore) were blocked with 5% (*w/v*) skim milk (232100, BD Bioscience, San Jose, CA, USA) in 0.1% Tween 20/PBS and incubated at 4 °C overnight in primary antibodies, which were diluted in blocking solution. Primary antibodies consisted of: mouse anti-OMA1 monoclonal (SC-515788, 1:200, Santa Cruz Biotechnology), rabbit anti-DELE1 polyclonal (21904-1-AP, 1:500, Proteintech), rabbit anti-PKR monoclonal (ab184257, 1:1000, Abcam), rabbit anti-ATF4 polyclonal (10835-1-AP, 1:1000, Proteintech), rabbit anti-DRP1 monoclonal (ab184247, 1:1000, Abcam), mouse or rabbit anti-OPA1 (612606, 1:1000, BD Bioscience; ab157457, 1:1000, Abcam), and rabbit anti-YME1L1 polyclonal (11510-1-AP, 1:1000, Proteintech). The following secondary antibodies were used: anti-mouse IgG, HRP-linked antibody (7076S, 1:5000; Cell Signaling) or anti-rabbit IgG, HRP-linked antibody (7074S, 1:5000; Cell Signaling). Relative changes in protein expression were calculated in relation to nonischemic retinas and expressed as the fold change.

### 2.6. OMA1 Activity Assay

The OMA1 activity assay used 8 peptides from the S1 cleavage sequence of OPA1 [40]. These 8 peptides contained a fluorescent MCA portion at the N-terminus and a DNP quencher portion at the C-terminus (custom synthesized by GeneScript, Nanjing, China). The following were rapidly added to a black opaque 96-well plate: (1) OMA1 activity assay buffer (50 mM Tris/HCl, pH 7.5, 40 mM KCl), (2) 5 μg protein sample, (3) ±200 μM zinc chelating agent N, N, N′, N′-tetra(2-pyridylmethyl)ethylenediamine (TPEN), and (4) OPA1 fluorescent reporter substrate (5 μM final concentration), at a final reaction volume of 100 μL per well. A multifunctional microplate reader (Biotek Synergy H1) was used to record the relative fluorescence (RFU) for 2 h (excitation/emission of 320/405 nm) every 5 min at 37 °C.

### 2.7. Transmission Electron Microscope

Retina samples were collected and fixed in 2.5% glutaraldehyde (G5882, Sigma-Aldrich) and 4% formaldehyde (P804536, Macklin) in 0.1 M HEPES buffer for 4 to 5 h. After washing in 0.1 M phosphate buffer three times, tissues were fixed with 1% osmic acid at 4 °C for 2 h. Tissues were washed in 0.1 M phosphate buffer three times, dehydrated using a graded alcohol series, and embedded in epoxy resin. Samples were examined under a Tecnai G2 Spirit electron microscope (FEI).

### 2.8. Flow-Cytometry Sorting of RGCs

RGC sorting was performed as previously described [41]. The retinal tissue was dissected 3 d after injury using sterile instruments. The enzymatically digested tissue was prepared into a cell suspension, before a fluorescently labeled antibody mixture was added, and a flow sorter was used to screen out CD90.2^+^CD48^neg^CD15^neg^CD57^neg^ cells. Sorted cells were subjected to immunofluorescence detection of RGC marker RBPMS (GTX118619, GeneTex, Irvine, CA, USA) for cellular immunophenotyping.

### 2.9. RNA-Seq Analysis

After RGCs were sorted by flow cytometry, total RNA was extracted from samples using TRIzol (Invitrogen) according to the method described by Chomczynski et al. [42]. DNA digestion was carried out after RNA extraction using DNaseI. RNA quality was determined by examining A260/A280 with a Nanodrop^TM^ One C spectrophotometer (Thermo Fisher Scientific Inc.). RNA integrity was confirmed by 1.5% agarose gel electrophoresis. Qualifying RNAs were quantified using a Qubit3.0 with a Qubit^TM^ RNA Broad Range Assay kit (Life Technologies, Carlsbad, CA, USA). Total RNA (2 μg) was used for stranded RNA sequencing library preparation using a KC-Digital^TM^ Stranded mRNA Library Prep Kit for Illumina^®^ (Catalog No. DR08502, Seqhealth Technology Co., Ltd. Wuhan, China) according to the manufacturer’s instructions. The kit eliminates duplication bias during the PCR and sequencing steps by employing unique molecular identifiers (UMIs) containing eight random bases to label the pre-amplified cDNA molecules. Library products containing 200–500 bps were enriched, quantified, and finally sequenced on an Illumina Novaseq 6000 platform. Sequence reads were mapped to the mouse genome assembly (GRCm38/mm10) using STAR v2.5.3a and differentially expressed genes (DEGs) were identified using edgeR package v3.12.1 The FDR cutoff was set at 0.05, and the minimum fold change was 1.5. We then generated a heatmap of differentially expressed genes using TBtools.

### 2.10. Statistical Analysis

All tissue processing, quantification, and data analysis were performed blindly throughout the study. Biological replicates (n) were based on accepted standards in the literature and prior experience from our laboratory. Parametric tests (ANOVA with Bonferroni post hoc test or unpaired two-tailed Student’s *t*-test) were used to determine the normality of data distribution (GraphPad Prism). Data are presented as the mean ± SEM. Statistical significance was set at *p* < 0.05.

## 3. Results

### 3.1. OMA1 Is Upregulated in the Inner Retina after Optic Nerve Injury

It is known that both Zn^2+^ and mitochondrial dysfunction contribute to progressive RGC loss following optic nerve injury [11]. The mitochondrial zinc metalloprotease OMA1 senses mitochondrial stress and regulates mitochondrial homeostasis [43]. Based on this, we investigated the relationship between OMA1 expression and optic nerve injury. Most of our studies have used the mouse optic nerve crush (ONC) model, which mainly causes changes in the inner retina as an experimental retina injury model [44]. Along with the experimental models, normal intact retinas with staining control were used throughout the study as a reference. First, we evaluated the expression levels of OMA1. We detected only modest levels of OMA1 in normal inner retinas. However, 1 d after ONC, we observed that the levels of OMA1 were markedly elevated in the inner nuclear layer (INL), inner plexiform layer (IPL), and ganglion cell layer (GCL), and continued to rise until day 3 (1.46 ± 0.06-fold above baseline in INL; 1.35 ± 0.10-fold above baseline in IPL; 2.20 ± 0.09-fold above baseline in βIII tubulin-positive RGC) (Figure 1C–F).

We also observed that OMA1 immunostaining was particularly prominent in the innermost INL and IPL on day 1 and was most pronounced in βIII tubulin-positive RGCs on day 3. Conversely, OMA1 staining in the INL and IPL declined to near and even below baseline levels 3–5 d after ONC, with RGCs within the GCL still retaining relatively strong staining on day 5 (1.73 ± 0.05-fold above baseline) (Figure 1C–F). The innermost INL is mainly composed of ACs, while the IPL primarily consists of axons of retinal interneurons and dendrites of RGCs. We detected an elevation in the levels of OMA1 first in the INL and IPL, and then in the GCL. This phenomenon is consistent with Zn^2+^ transfer from ACs to RGCs after ONC [3].

### 3.2. Retinal Cellular Localization of OMA1

**Figure 2:**
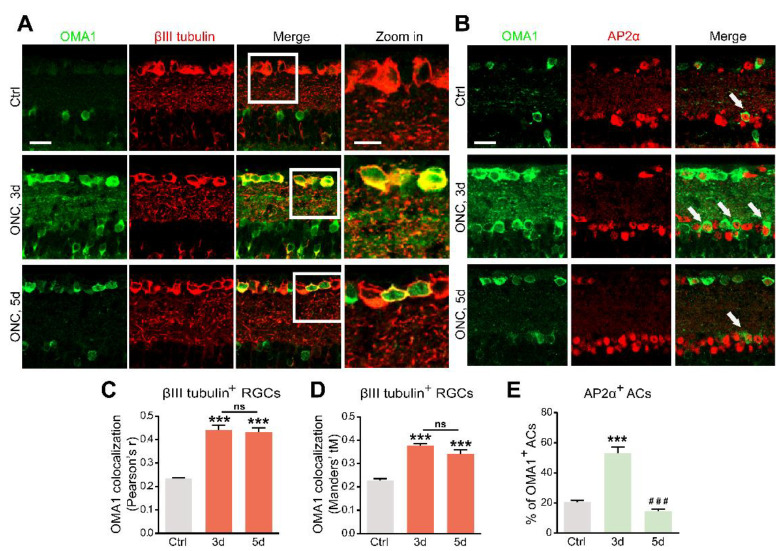
Upregulation of OMA1 in ACs and RGCs: (**A**). Images of retinal cross-sections immunostained for OMA1 and βIII tubulin to visualize the expression of OMA1 in RGCs before ONC and 3 and 5 d post-ONC; (right) enlargements of framed areas. Scale bars, 20 μm (lower magnification), 10 μm (higher magnification). (**B**). Double-immunostaining for OMA1 and the amacrine cell marker AP2α to visualize OMA1-positive ACs in INL before ONC and 3 and 5 d post-ONC. Scale bar, 20 μm. (**C**,**D**). Quantitative analysis using Pearson’s r value and Mander’s value, tM showed a significantly higher colocalization of OMA1 with RGCs after ONC. n = 3, 6, 6. One way ANOVA, *** *p* < 0.001 compared with uncrushed controls; ns, no significance, compared with 3 d post-ONC. (**E**). Quantitation of the percentage of OMA1-positive ACs in INL before ONC and 3 and 5 d post-ONC revealed higher colocalization of OMA1 with ACs on 3 d after ONC, but reduction to baseline level 5 d after ONC. n = 3, 5, 5. One-way ANOVA, Bonferroni post hoc tests, *** *p* < 0.001 compared with uncrushed controls, ### *p* < 0.001 compared with 3 d post-ONC. All bars show the mean ± SEM.

The OMA1 trends in the inner retina parallel the transsynaptic process of Zn^2+^ in ACs and RGCs, raising the question of cell-type specificity. We analyzed the colocalization and cell-type specificity of OMA1 in the inner retina. Double-immunostaining revealed weak overlap in normal retinas, and substantial overlap on days 3 and 5 between OMA1 and the RGC marker βIII tubulin (Figure 2A,C,D) in ONC models. We used the pan-amacrine cell marker AP2α to visualize the nuclei of ACs in the INL. Cells with AP2α-positive nuclear and OMA1-positive cytoplasmic staining were defined as OMA1^+^ ACs. We found that the percentage of OMA1^+^ ACs was increased from 20.60 ± 1.01% in normal retinas to 53.19 ± 3.82% on day 3 in ONC retinas, before decreasing to 14.40 ± 1.59% on day 5 (Figure 2B,E). The data confirmed that levels of OMA1 in ACs decreased remarkably following the peak on day 3. However, OMA1 expression levels in RGCs remained relatively high.

**Figure 3:**
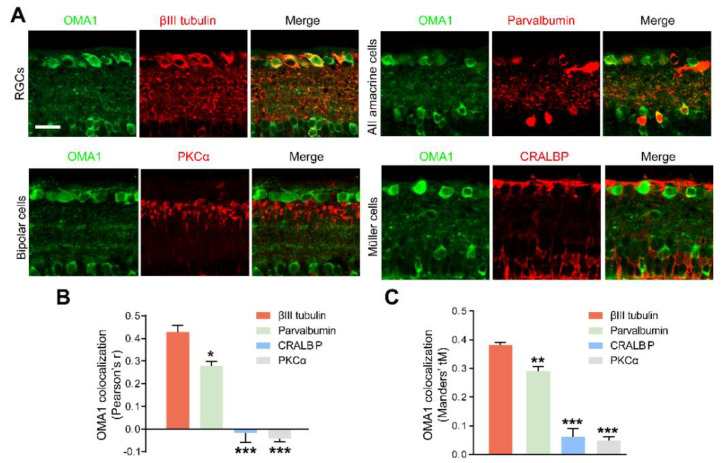
Retinal cellular localization of OMA1: (**A**). Confocal images of retinal cross-sections show overlap of OMA1 with RGC marker βIII tubulin and amacrine cell marker parvalbumin, but not with bipolar cell marker PKCα or Müller cell marker CRALBP. Scale bar, 25 μm. (**B**,**C**). Quantitative Pearson’s r value and Mander’s value, tM of OMA1 showed a significantly higher colocalization of OMA1 with RGCs and ACs than either bipolar or Müller cells; n = 3 retinas per group; one-way ANOVA, Bonferroni’s post hoc test, *** *p* < 0.001, ** *p* < 0.01, * *p* < 0.05, compared with βIII tubulin. All bars show the mean ± SEM.

In addition, we detected that OMA1 showed a strong overlap with the calcium-binding protein parvalbumin, a marker for AII amacrine cells, which are a unique subtype of ACs (Figure 3A). However, we noticed that OMA1 rarely overlapped with the Müller cell marker cellular retinaldehyde-binding protein (CRALBP) or bipolar cell synapse marker protein kinase C-α (PKCα) (Figure 3A–C). The findings indicate that OMA1 was primarily distributed in ACs and RGCs, but not in other retinal interneurons and glial cells, suggesting the critical role of OMA1 in the AC-RGC regulatory network.

Our results showed that OMA1 was first upregulated mainly in the soma and processes of ACs, and subsequently appeared and maintained within RGCs, following the pattern of Zn^2+^ transfer [3]. Given the cell-type specific evidence for the similarity in regulation patterns (including chronological specificity) between Zn^2+^ and OMA1, we speculated that OMA1 upregulation is mediated by the AC-to-RGC transfer of Zn^2+^ following optic nerve injury.

### 3.3. Zn^2+^ Mediates OMA1 Upregulation and Mitochondrial Damage

**Figure 4:**
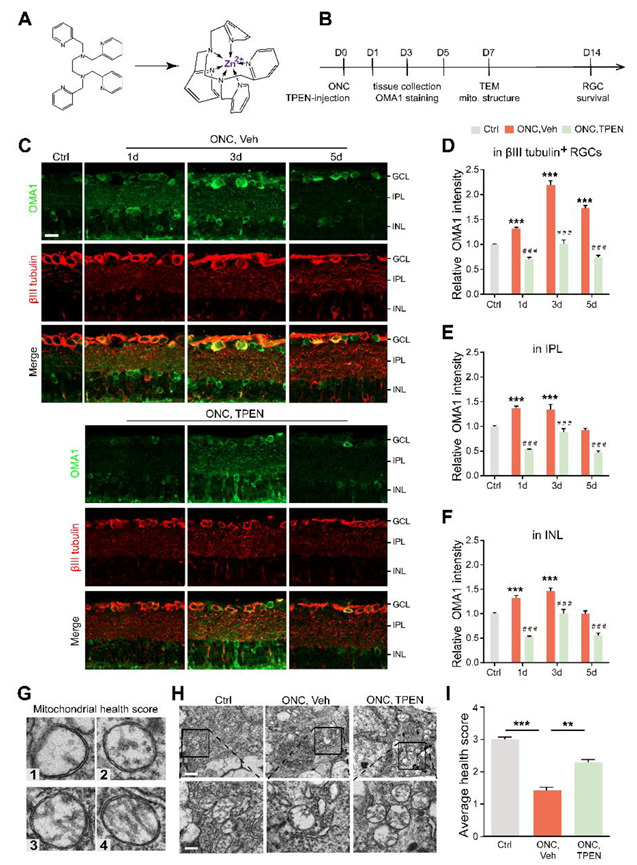
Chelating Zn^2+^ by TPEN decreased OMA1 expression and attenuated mitochondrial damage: (**A**). The chemical structures of TPEN and with chelated Zn^2+^. (**B**). Timeline for ONC and TPEN treatment. (**C**). OMA1 immunostaining in retinas of wild-type mice with and without TPEN treatment. n = 7 (Ctrl); n = 6 (ONC, Vehicle); n = 7 (ONC, TPEN). Scale bar, 20 μm. (**D**–**F**). Quantification of OMA1 expression in βIII tubulin-positive RGCs, IPL, and INL at indicated time points after ONC with 100 μM TPEN. One-way ANOVA, Bonferroni post hoc tests, *** *p* < 0.001 compared with normal retina controls; Two-way ANOVA with Bonferroni’s post hoc test ### *p* < 0.001, decrease compared with vehicle-treated controls at the same time point. Values are normalized to normal control retinas. (**G**). Mitochondrial health scale with 4 representative transmission electron microscopy (TEM) images of mitochondria in RGCs and corresponding scores. (**H**). Representative TEM images in RGCs from different experimental groups. Scale bars, 1 μm (lower magnification), 500 nm (higher magnification). (**I**). Based on the mitochondrial health scale, 20–30 mitochondria per retina were scored in 1–4. n = 4. One-way ANOVA with Bonferroni’s post hoc test, *** *p* < 0.001, ** *p* < 0.01. All bars show the mean ± SEM.

To further investigate whether the level of OMA1 was regulated by Zn^2+^ and evaluate the effects of pharmacologically suppressing Zn^2+^ on retinal mitochondria, we examined whether Zn^2+^ chelators can downregulate OMA1 and affect mitochondrial structure when administered after ONC. To this end, we performed an intraocular injection of the high-affinity, membrane-permeable Zn^2+^ chelator TPEN (N,N,N′,N′-tetrakis (2-pyridylmethyl) ethylenediamine) immediately after ONC and analyzed the level of OMA1 compared with that in controls receiving vehicle injections (Figure 4A,B). TPEN decreased expression levels of OMA1 to near or below baseline levels on days 1, 3, and 5 (Figure 4C–F). We further observed that the TPEN-induced downregulation of OMA1 was similar in the INL and IPL, and was more significant in βIII tubulin-positive RGCs within the GCL (OMA1 decreased from 2.20 ± 0.09-fold above baseline to 1.02 ± 0.07-fold by day 3), which was consistent with the permeability and Zn^2+^ elimination effect of TPEN in the retina (Figure 4C–F). Moreover, after TPEN injection, OMA1 staining still showed a peak on day 3 and relatively lower levels on days 1 and 5, which indicated that the TPEN-induced downregulation of OMA1 was mediated by chelating Zn^2+^.

Studies have shown that the Zn^2+^ overload of mitochondria leads to mitochondrial structural damage [16,23]. To determine the relationship between Zn^2+^ and RGC mitochondrial damage during optic nerve injury, we examined RGC mitochondria using transmission electron microscopy (TEM). We applied a mitochondrial health scoring system based primarily on cristae appearance, then selected and scored four representative mitochondria (Figure 4G). A higher score indicated a more preserved mitochondrial structure. Accordingly, 7 d after ONC, representative TEM images showed severe swelling, reduced matrix density, cristae rupture, and the disappearance of inner and outer membrane integrity of RGC mitochondria (Figure 4H). However, we noticed that the TPEN-treated group had higher mitochondrial health scores compared with the vehicle-treated controls (Figure 4H,I). In addition, using immunostaining of TOMM20 and mitochondrial network analysis, we found the ratio of network/individual (networks = healthy, individuals = unhealthy) decreased after ONC, and could be restored by TPEN-treatment (Figure S1A,B). These findings indicated that Zn^2+^ chelation alleviated ONC-induced mitochondrial disruption.

Overall, after optic nerve injury, Zn^2+^ upregulates OMA1 in the inner retina and mediates RGC mitochondrial damage. Early chelation of Zn^2+^ inhibits OMA1 and protects RGC mitochondria, suggesting that this is the long-term mechanism of RGC protection by TPEN.

### 3.4. Blocking the Transsynaptic Process of Zn^2+^ Decreases OMA1 and Alleviates Mitochondrial Structural Damage

**Figure 5:**
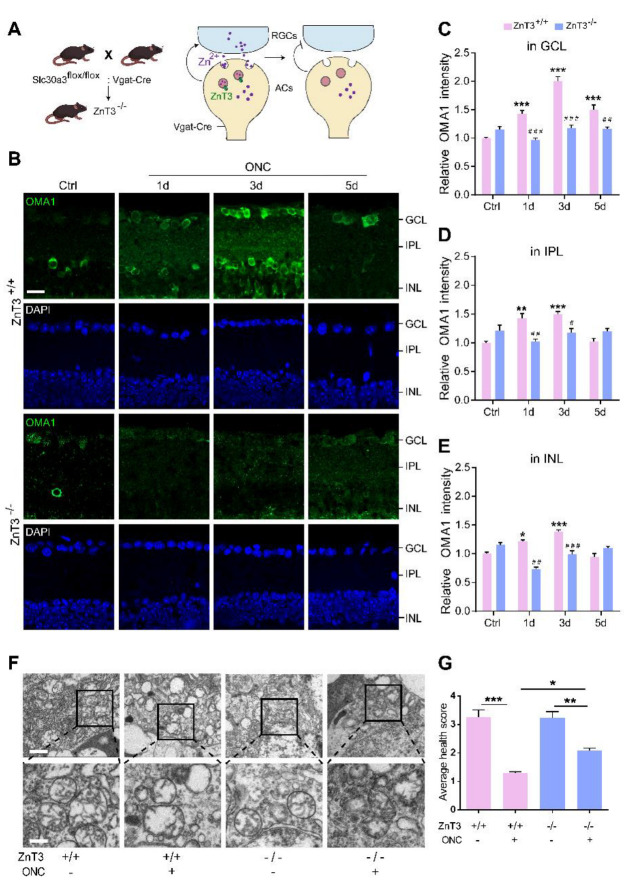
Inhibiting Zn^2+^ transfer with ZnT-3 deletion decreased OMA1 and attenuated mitochondrial damage: (**A**). Schematic of Zn^2+^ transfer via ZnT-3-containing vesicles in amacrine cells (ACs) showing that Vgat-Cre mice selectively express Cre in ACs. ZnT-3 (encoded by Slc30a3) deletion was achieved using the Cre-LoxP system only in ACs to eliminate vesicular Zn^2+^. (**B**). OMA1 immunostaining in the retinas of ZnT-3^+/+^ and ZnT-3^−/−^ littermates. Scale bar, 20 μm. (**C**–**E**). Quantification of OMA1 expression levels in the GCL, IPL, and INL of ZnT-3^−/−^ and ZnT-3^+/+^ littermates. Two-way ANOVA with Bonferroni’s post hoc test, *** *p* < 0.001 compared with normal retina controls; ### *p* < 0.001, ## *p* < 0.01, # *p* < 0.05, decrease compared with the ZnT-3^+/+^ group at the same time point. (**F**). Transmission electron microscopy (TEM) images of RGC mitochondria in ZnT-3^+/+^ and ZnT-3^−/−^ littermates. Scale bars, 1 μm (lower magnification), 500 nm (higher magnification). (**G**). Based on the mitochondrial health scale, 20–30 mitochondria per retina were scored 1–4. n = 4. One-way ANOVA with Bonferroni’s post hoc test, *** *p* < 0.001, ** *p* < 0.01, and * *p* < 0.05. All bars show the mean ± SEM.

Our previous studies demonstrated that the Zn^2+^ transporter ZnT-3 (encoded by Slc30a3) loads Zn^2+^ into the synaptic vesicles of ACs, resulting in Zn^2+^ accumulation in GCL cells [3]. Here, we mated Slc30a3^fl/fl^ mice with mice expressing Cre recombinase under the control of the Vgat promoter (specifically active in retinal ACs) [45]. The homozygote offspring (ZnT-3^−/−^) that lack ZnT-3 in ACs blocked AC-to-RGC transfer of Zn^2+^, partially eliminating its accumulation in RGCs (Figure 5A).

Immunostaining revealed that deletion of ZnT-3 in ACs did not affect the expression of OMA1 in mice without ONC, but markedly suppressed OMA1 in INL, IPL, and GCL on days 1 and 3 after ONC. However, we observed that 5 d after ONC, the level of OMA1 in the retina of ZnT-3^−/−^ mice was not significantly different compared with that in wild-type INL and IPL, but sustained the suppressive effect in GCL (decreased from 1.50 ± 0.09-fold above baseline to 1.16 ± 0.04-fold, two-way ANOVA, Bonferroni-corrected *p* = 0.0058) (Figure 5B–E). In addition, unlike after TPEN injection, the level of expression of OMA1 in ZnT-3^−/−^ mice did not peak on day 3, but instead showed a persistent increase over 0–5 d after ONC. These findings suggested that partially eliminating Zn^2+^ accumulation in RGCs by blocking the synaptic transfer of Zn^2+^ decreased the early expression of OMA1. However, in later stages of optic nerve injury, Zn^2+^ accumulation in interneurons sustained upregulation of OMA1 in INL and IPL, but left the postsynaptic RGC unaffected. Consistently, we noticed that a week after ONC, the mitochondrial health scores of RGCs in ZnT-3^−/−^ mice were somewhat higher than those of their ZnT-3^+/+^ littermates (Figure 5F,G).

Thus, the early inhibition of Zn^2+^ by Zn^2+^ chelation and transfer blockage downregulate OMA1 in the inner retina and protect RGC mitochondria, suggesting that overexpression of OMA1 and mitochondrial damage are mediated by Zn^2+^.

### 3.5. Zn^2+^ Increases OMA1 Activity

**Figure 6:**
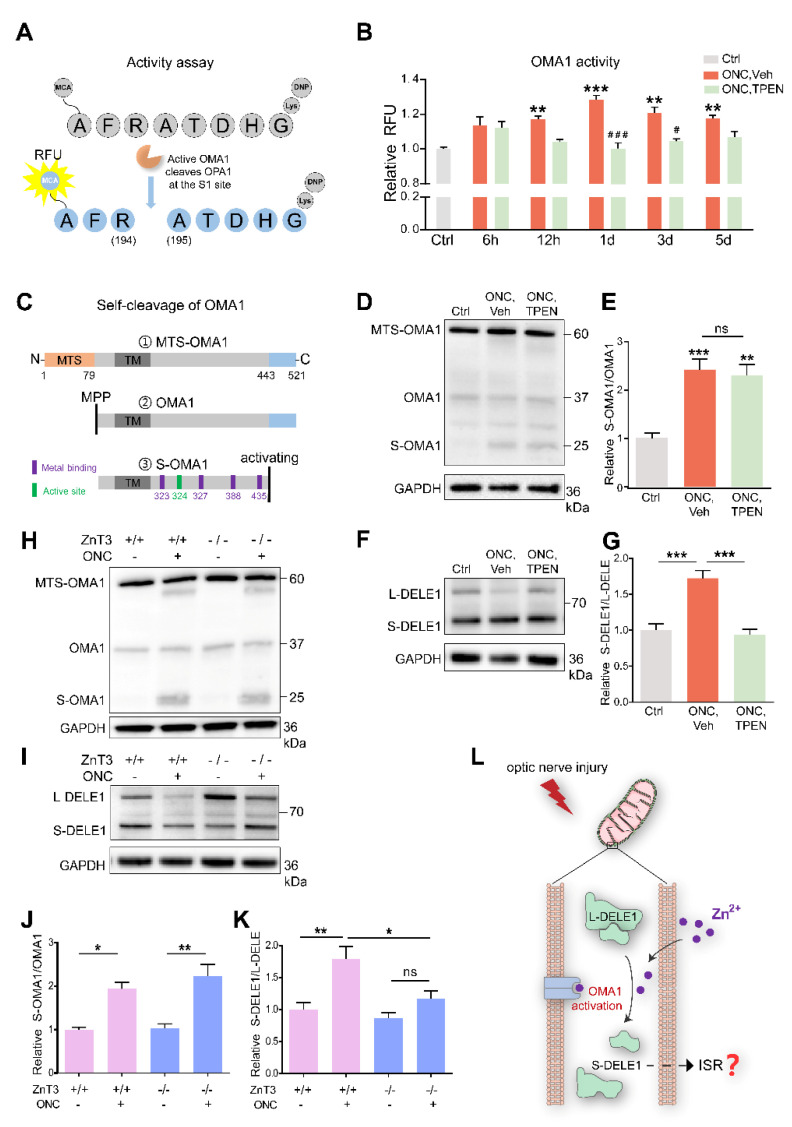
Zn^2+^ increased OMA1 activity and S-DELE1 accumulation in retina: (**A**) Schematic of OMA1 activity fluorescence-based assay. MCA, 7 methoxycoumarin 4 ylacetyl; DNP, 2,4 dinitrophenyl. (**B**) Activity of OMA1 in retina before and after ONC at indicated time points with 100 μM TPEN treatment. n = 3 (Ctrl); n = 3, 4, 4, 4, 4 (ONC, Vehicle); n = 3, 3, 4, 4, 4 (ONC, TPEN). One-way ANOVA with Bonferroni’s post hoc tests, *** *p* < 0.001, ** *p* < 0.01, * *p* < 0.05, compared with normal retina controls; Two-way ANOVA with Bonferroni’s post hoc test ### *p* < 0.001, # *p* < 0.05, decreased compared with vehicle-treated controls at the same time point. Values are normalized to normal control retinas. (**C**) Schematic of the OMA1 turnover process showing cleavage, metal binding, and active sites. MTS, mitochondrial targeting sequences; TM, transmembrane domains; MPP, mitochondrial processing peptidases. (**D**) Western blotting of lysates from retinas using primary antibodies against OMA1 and GAPDH (loading control). (**E**) The relative ratios of S-OMA1/OMA1 bands were quantified and normalized to normal controls. n = 3. One-way ANOVA with Bonferroni’s post hoc tests, *** *p* < 0.001, ** *p* < 0.01, compared with normal retina controls, ns, no significance. (**F**) Western blotting of lysates from retinas using primary antibodies against DELE1 and GAPDH (loading control). (**G**) The relative ratios of S-DELE1/L-DELE1 bands were quantified and normalized to normal controls. n = 3. One-way ANOVA, Bonferroni’s post hoc tests, *** *p* < 0.001. (**H**) Western blotting of retinal lysates from ZnT-3^−/−^ and ZnT-3^+/+^ littermates using primary antibodies against OMA1 and GAPDH (loading control). (**I**) Western blotting of retinal lysates from ZnT-3^−/−^ and ZnT-3^+/+^ littermates using primary antibodies against DELE1 and GAPDH (loading control). (**J**) Quantification of relative S-OMA1/OMA1 band intensities normalized to ZnT-3^+/+^ normal retina controls, n = 4. One-way ANOVA with Bonferroni’s post hoc tests, ** *p* < 0.01, * *p* < 0.05. (**K**) Quantification of relative S-DELE1/L-DELE1 band intensities normalized to ZnT-3^+/+^ normal retina controls, n = 4. One-way ANOVA with Bonferroni’s post hoc tests, ** *p* < 0.01, * *p* < 0.05; ns, no significance. (**L**) Model for mechanism by which Zn^2+^ induces activation of OMA1 and cleavage of DELE1 in optic nerve injury. All bars show the mean ± SEM.

As immunostaining only reflects the level of expression but not the proteolytic activity of OMA1, we sought to determine whether OMA1 direct activity was changed under injury conditions. The canonical biological function of OMA1 is to cleave OPA1 at the S1 site [46]. According to the first direct activity assay for OMA1 reported by Tobacyk et al. [40], we synthesized an eight-amino acid peptide based on the OPA1 S1 cleavage site sequence (MCA-AFRATDHG-(lys)DNP) containing a 7-methoxycoumarin-4-ylacetyl (MCA) fluorophore moiety on the N-terminus and a 2,4-dinitrophenyl (DNP) quencher moiety on the C-terminus. This MCA moiety on the fluorogenic reporter substrate has very low intrinsic fluorescence due to the proximity of the DNP quencher group. Once OMA1 cleaves the S1 site, which is between the 194 arginine (R) and 195 alanine (A) residues, the DNP quencher is removed, allowing MCA fluorescence to reflect the OMA1 activity (Figure 6A).

We detected that the retinal OMA1 activity started to increase at 6–12 h after ONC and continued to rise over the next 12 h. Subsequently, over 1–5 d, the proteolytic activity of OMA1 partially declined but remained relatively increased compared with that in normal retina. We further observed that administration of the Zn^2+^ chelator TPEN immediately after ONC exerted an inhibitory effect at 12 h and significantly inhibited the activity of OMA1 on days 1 and 3 (Figure 6B), consistent with the Zn^2+^ elimination effect [3]. We examined the effect of inorganic Zn^2+^ on the activation of OMA1 by injecting 1 mM ZnCl_2_ intraocularly. We found that inorganic Zn^2+^ failed to increase the activity of OMA1 (Figure S1C), in line with previous results that ZnCl_2_ did not impair RGC survival or increase Zn^2+^ signaling in the retina [3]. This probably results from inorganic Zn^2+^ being unable to penetrate the internal limiting membrane of the retina.

Before the direct activity assay for OMA1 was reported, the gold standard for qualitatively assessing OMA1 activity was Western blot analysis of the OPA1 cleavage (long to short form) [47,48]. We thus detected endogenous retinal expression of OPA1 using Western blotting. We found that on day 1 after ONC, the ratio of soluble short-form (S-) OPA1 to membrane-anchored long-form (L-) OPA1 was increased and TPEN injection inhibited this process (Figure S1D,E). Thus, the findings indicate that ONC-induced Zn^2+^ accumulation stimulated the protease activity of retinal OMA1, which could be measured using an S1 cleavage site-based fluorogenic reporter system.

In addition, we noticed that neither ONC nor TPEN affected the level of expression of another zinc metalloproteinase, YME1L, which is an ATP-dependent i-AAA protease, nor did they affect the expression of retinal mitochondrial fission factor dynamin-related protein 1 (DRP1) (Figure S1D,F,G). These findings indicated that after ONC, Zn^2+^ mediated RGC mitochondrial damage by activating OMA1 but not YME1L1, and by decreasing fusion factor OPA1, but not DRP1.

After translation and transfer to mitochondria, OMA1 is cleaved at its N-terminus by mitochondrial processing peptidases (MPP) to remove the mitochondrial targeting sequence (MTS) to enter mitochondria [49]. Upon mitochondrial stress stimuli, self-cleavage at its C-terminus activates the proteolytic function of OMA1 [50]. After self-cleavage, S-OMA1 retains its active and zinc-binding sites, consistent with the Zn^2+^-dependent character of OMA1 (Figure 6C). The dynamic process of OMA1 turnover partially reflects its activity. Our Western blot assay revealed the presence of MTS-OMA1 (~60 kDa), OMA1 (~37 kDa), S-OMA1 (~25 kDa), and an undefined form (~30 kDa). We found that ONC increased the ratio of S-OMA1/OMA1 on day 3, indicating an increase in self-cleavage and activation of OMA1 (Figure 6D). However, neither TPEN injection nor ZnT-3 deletion affected this turnover process (Figure 6D,E,H,J).

Combining our data on the direct activity and turnover of OMA1, we speculated that TPEN directly chelated Zn^2+^ to inhibit the activity of OMA1 without affecting its turnover.

### 3.6. Zn^2+^ Increases Cleavage of DELE1

Under mitochondrial stress, OMA1 cleaves long-form (L-) DELE1 to short-form (S-) DELE1. The OMA1-DELE1 signal links mitochondrial stress to cytoplasmic ISR, which is conserved across different cell types, including neurons [28,29,51]. Therefore, we performed retinal DELE1 analysis using Western blotting. Our quantitative results showed that the ratio of S-DELE1/L-DELE1 was markedly increased 3 d after ONC. Interestingly, TPEN injection decreased the S-DELE1/L-DELE1 ratio to baseline levels (Figure 6F,G). Consistently, we found that ZnT-3 deletion partially recovered DELE1 cleavage (Figure 6I,K). These findings suggest that ONC-induced Zn^2+^ stimulates cleavage of DELE1. Thus, we demonstrated that Zn^2+^ leads to the upregulation and activation of OMA1 following optic nerve injury, which then mediates the cleavage of DELE1 into a short form (Figure 6L).

### 3.7. Zn^2+^–OMA1–DELE1 Axis Relays to the Integrated Stress Response in RGCs

**Figure 7:**
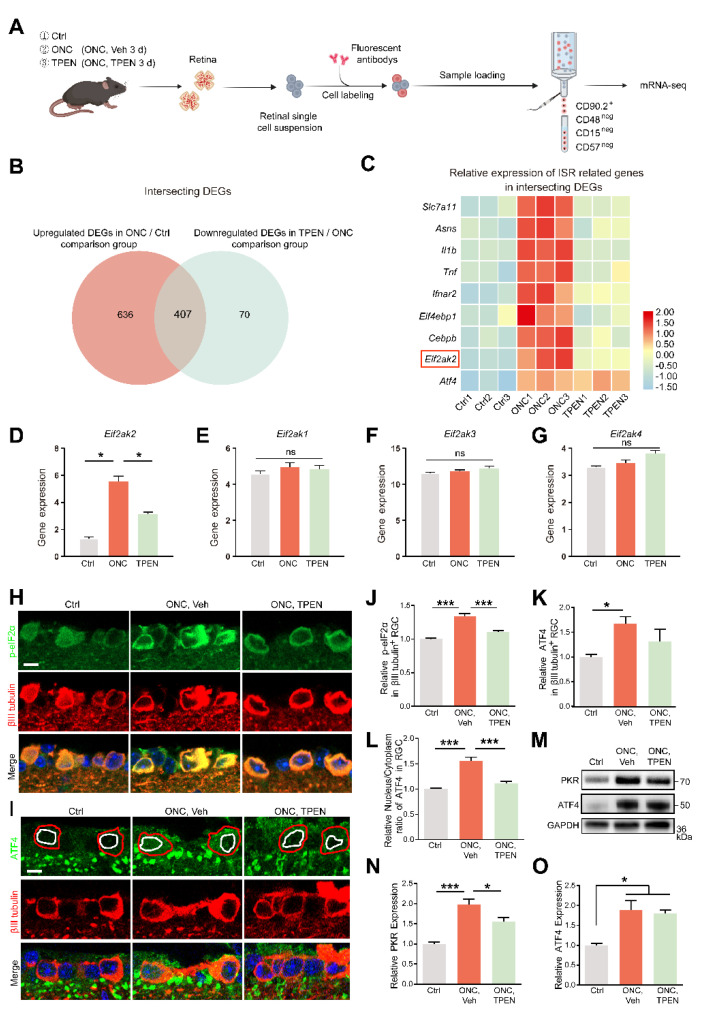
Zn^2+^-induced mitochondrial signals are relayed to ISR in RGCs: (**A**). Schematic of the experimental design for RGC mRNA sequencing. Retinas were harvested from wild-type mice with and without TPEN treatment. Each group included three libraries. (**B**). Integrated comparative analysis of upregulated DEGs in ONC/Ctrl and downregulated DEGs in TPEN/ONC, showing the intersecting genes. (**C**). Representative ISR-related genes were enriched in the intersecting genes. (**D**–**G**). The gene levels of Eif2ak1-4 were quantified. One-way ANOVA with Bonferroni’s post hoc tests, * *p* < 0.05; ns, no significance. (**H**). p-eIF2α and βIII tubulin immunofluorescence staining in control and 5 d post-optic nerve injury mouse retinas. Scale bar, 10 μm. (**I**). ATF4 and βIII tubulin immunofluorescence staining in control and 5 d post-optic nerve injury mouse retinas. Red circles show the areas of RGCs, whereas white circles show the areas of nuclei. Scale bar, 10 μm. (**J**,**K**). Quantitation of the expression of p-eIF2α, n = 6, 5, 4 and ATF4, n = 5, 7, 5. in βIII tubulin-positive RGCs (normalized to normal control). One-way ANOVA with Bonferroni’s post hoc tests, *** *p* < 0.001, * *p* < 0.05. (**L**). Quantification of the nuclear/cytoplasm ratio of distribution of ATF4 in βIII tubulin-positive RGCs (normalized to normal control). n = 5, 7, 4. One-way ANOVA with Bonferroni’s post hoc tests, *** *p* < 0.001. (**M**–**O**). Western blotting and quantification of the expression of PKR and ATF4 in control and 5 d post-optic nerve injury mouse retinas (normalized to normal control). n = 3. One-way ANOVA with Bonferroni’s post hoc tests, *** *p* < 0.001, * *p* < 0.05. All bars show the mean ± SEM.

To identify whether ISR is the downstream signaling pathway of DELE1 cleavage, we conducted mRNA sequencing of flow-cytometry-isolated RGCs 3 d after ONC (Figure 7A and Figure S2A). We then identified differentially expressed genes (DEGs) in each of these treated RGCs using a cutoff of at least 1.5-fold change and FDR of at least 0.05.

We focused on the intersecting genes that were upregulated after ONC (ONC/Ctrl; fold change > 1.5; *p* < 0.05) and recovered in TPEN (TPEN/ONC; fold change < −1.5; *p* < 0.05), namely, TPEN target genes (Figure 7B). Using a signature of mitochondrial ISR-related genes curated from the literature [28,52,53,54,55], we confirmed the significant enrichment of ISR-induced inflammatory mediators (e.g., Il1b and Tnf), eif2α-phosphorylation factors (e.g., Eif2ak2), and ATF4 targets (e.g., Slc7a11, Asns, and Cebpb) with intersecting genes (Figure 7C). Of note, the phosphorylation of eIF2α is mediated by four eIF2α kinases (HRI, PKR, PERK, and GCN2) encoded by Eif2ak1-4. Our transcriptome data demonstrated that ONC and TPEN treatment did not affect the activity of Eif2ak1, 3, and 4, strongly suggesting that TPEN treatment inhibited the phosphorylation of eIF2α and activation of ISR by downregulating Eif2ak2 specifically (Figure 7D–G).

We also investigated the protein levels of PKR (encoded by Eif2ak2), phospho-eIF2α (p-eif2α), and ATF4. Consistently, we observed an increase in the levels of PKR and p-eIF2α in ONC retinas on day 5 compared with those in normal retinas. We also found that TPEN treatment restored the levels of PKR and p-eIF2α (Figure 7H,J,M,N). However, using RNA-seq, immunofluorescence assay, and Western blotting, we noticed that the transcriptomic and protein levels of ATF4 in RGCs of the TPEN group were unaltered compared with those in the vehicle-treated controls (Figure 7C,I,K,M,O). Upon activation of ISR, ATF4 reprograms translation via cytoplasm-to-nuclear translocation. Therefore, we further analyzed the subcellular distribution of ATF4 in RGCs. We found that the nucleus/cytoplasm ratio of ATF4 was increased 5 d after ONC but decreased by TPEN treatment (Figure 7I,L). These findings confirmed that the Zn^2+^-induced activation of OMA1 and accumulation of S-DELE1 led to an ISR in RGCs, likely via the upregulation of PKR, which TPEN treatment significantly inhibited.

### 3.8. Zn^2+^-OMA1 and ISR Act in the Same Signaling Pathway to Regulate RGC Survival

**Figure 8:**
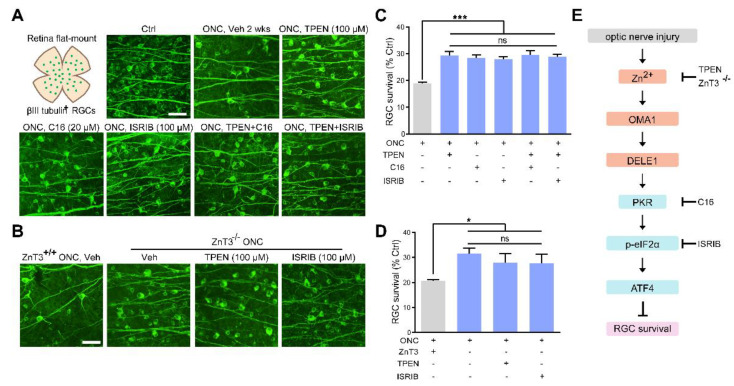
Zn^2+^ elimination and ISR inhibition promoted RGC survival: (**A**,**B**). Retinal flat mounts immunostained for βIII tubulin to visualize RGCs in 2 w post-ONC normal control mice with pharmacological or genetic treatment as indicated. Scale bar, 50 μm. (**C**,**D**). Quantitative results. n = 4. One-way ANOVA and Bonferroni’s post hoc tests. *** *p* < 0.001, * *p* < 0.05 compared with vehicle-treated controls; ns, no significance. (**E**). Schematic of the Zn^2+^-induced OMA1–DELE1–PKR–ISR axis in optic nerve injury and therapeutic targets.

We next addressed whether the Zn^2+^-OMA1-PKR-ISR pathway participates in RGC death after ONC. In line with our previous report [3], we confirmed that intraocular injection of TPEN immediately after ONC significantly promoted RGC survival 14 d later when compared with controls receiving vehicle injection (Figure 8A,C). Moreover, we investigated the pro-RGC survival efficacy of ZnT-3 conditional knockout. Similar to the global knockout [3], ZnT-3 conditional knockout in ACs did not affect the basal number of RGCs (Figure S2B,C), but promoted RGC survival following optic nerve injury (Figure 8B,D).

We then examined the protective effect of C16, a PKR inhibitor, and ISRIB, a small-molecule ISR inhibitor at optimal concentrations (20 μM and 100 μM, respectively) (Figure S2D–G). We observed that ZnT-3 deletion, treatment with TPEN (100 μM), C16 (20 μM), or ISRIB (100 μM) did not result in any significant differences in RGC protection. Combining ZnT-3 deletion or TPEN injection with C16 or ISRIB did not augment the 2 w survival beyond the level achieved with either alone, highlighting that altered ISR and Zn^2+^ act in the same pathway to mediate RGC loss in ONC (Figure 8A–D). Our findings underlined the importance of Zn^2+^-OMA1 and PKR-induced ISR as primary mediators of RGC loss in the pathogenesis of optic nerve injury.

## 4. Discussion

In the current study, we investigated the downstream mechanisms of Zn^2+^ in postsynaptic RGC somatic injury and explored potential therapeutic targets for neuroprotection. Results of this study show that: (1) the structural damage of RGC mitochondria in optic nerve injury is related to increased levels of Zn^2+^; (2) the mitochondrial metallopeptidase OMA1 in the inner retina is activated by synaptic transfer of Zn^2+^; (3) Zn^2+^-induced OMA1 activation relays to ISR in RGCs; (4) Zn^2+^ elimination inhibits the OMA1-ISR pathway and attenuates RGC mitochondrial structural damage; and (5) both Zn^2+^ elimination and ISR inhibition promote the survival of RGCs after optic nerve injury. Collectively, these data suggest the involvement of Zn^2+^-OMA1-ISR signaling in Zn^2+^-induced RGC loss after optic nerve injury.

Recent studies have emphasized the essential role of Zn^2+^ in the central nervous system and retina [11,16,56]. Excessive levels of Zn^2+^ are implicated in nerve injury, and Zn^2+^ elimination results in marked neuron protection [3,5]. Mechanistically, impaired mitochondrial structure and function contribute to Zn^2+^-induced neuronal death [16,17]. However, the underlying molecular pathways remain poorly understood. The majority (~90 %) of total zinc in neurons is tightly bound to metalloenzymes, transcription factors, and other zinc-containing proteins, in which zinc serves as a cofactor for enzymatic activity or maintains the three-dimensional protein structure [57,58]. The remaining 10% of total zinc is called free or mobile zinc and is either present as hydrated ions or loosely bound to proteins. Metalloproteins have essential biological processes, and each of them must bind specific metal ions to function. The metal-binding affinities of natural proteins primarily follow the Irving–Williams series (Mn^2+^ <  Fe^2+^ < Co^2+^ < Ni^2+^ < Cu^2+^ > Zn^2+^) and the electronic properties of metal ions [59]. Accordingly, metalloproteins overwhelmingly tend to bind Zn^2+^ and Cu^2+^ [60]. When combining the protective effects of Zn^2+^ chelators in nerve injury, balance between Zn^2+^ and neuronal zinc metalloproteins is critical for mitochondrial homeostasis and cell survival. Therefore, the mitochondrial zinc metalloproteins could be downstream targets that mediate increased Zn^2+^ signaling in response to RGC damage in optic nerve injury.

Our study confirmed that structural damage to RGC mitochondria was related to increased levels of Zn^2+^. We found that the mitochondrial zinc-dependent metalloprotease OMA1 was tightly regulated by endogenous Zn^2+^ during optic nerve injury. Kaser et al. first identified OMA1 as a membrane-bound metallopeptidase that has activities overlapping with the m-AAA protease, which depends on unspecified divalent metal ions [19]. The zinc-binding motif of OMA1 was reported later [22,61]. Our results confirmed the essential role of Zn^2+^ in OMA1 activation during neuronal injury. In the retina, OMA1 is primarily located in presynaptic Zn^2+^ initial cells (ACs) and postsynaptic Zn^2+^ target cells (RGCs). After optic nerve injury, the retinal proteolytic activity of OMA1 rapidly increased, and its expression level was upregulated first in ACs (1 d) and then in RGCs (3 d and 5 d), following the transfer process of Zn^2+^. Elimination of Zn^2+^ by chelation or deletion of the zinc transporter ZnT-3 in ACs reversed the concomitant upregulation of OMA1 and preserved the mitochondrial structure in RGCs. These results suggest a previously unknown link between optic nerve injury and retinal expression of OMA1.

Inhibition of OMA1 or Zn^2+^ could promote neuron survival; however, the molecular mechanisms remain unclear [18,27,62,63]. Ablation of OMA1 has been reported to protect against heart failure in multiple mouse models of mitochondrial dysfunction [26,27]. In particular, the inhibition of OMA1 protects neurons against mitochondrial depolarization and cardiomyocytes against hypoxia-reperfusion injury [19,26,51]. Consistent with these findings, moderate overexpression of OPA1, the primary OMA1 proteolytic substrate identified thus far, protects RGCs and neurons in experimental glaucoma and ischemia-reperfusion models, respectively [64,65]. Notably, excessive OPA1 overexpression causes developmental arrest, limiting the therapeutic effects of OPA1 supplementation [66]. TPEN, via zinc chelation, exerts protective effects in various disease models, including neurodegeneration and hypoxia-reperfusion [18,67,68]. For diseases that benefit from Zn^2+^ chelation or OMA1 inhibition, our results suggest the crosstalk between Zn^2+^ and OMA1 dimerization is a potential molecular mechanism.

Recently, the cascade in which OMA1 relays mitochondrial stress signals to the cytoplasmic ISR via DELE1 cleavage and eIF2α kinase activation was reported to be conserved in several diseases, including neurodegenerative disorders [69,70,71]. In the present study, our results suggested that the OMA1–DELE1–ISR axis is also involved in optic nerve injury-induced RGC death attributed to Zn^2+^ accumulation. However, our RGC transcriptomic data identified differential activity of only Eif2ak2 (encoding PKR), but not the previously reported Eif2ak1, 3, 4 (encoding HRI, PERK, and GCN2, respectively), which were upregulated after ONC and reversed by Zn^2+^ chelation. PKR is the most recently evolved ISR kinase, with known roles in innate immunity and various neurological disorders [72]. More specifically, PKR detects viral and endogenous double-stranded RNAs (dsRNAs), including leaked mitochondrial transcripts and nuclear dsRNAs, and has a function in regulating ISR [73,74]. These results indicate that eIF2α kinases exert diverse functions under different stimuli, but similarly phosphorylate the eukaryotic translation initiation factor eIF2α to trigger ISR.

Both the inhibition and maintenance of ISR are potential therapeutic strategies [37,52,75,76,77]. Ablation of OMA1 was previously found to protect against heart failure in multiple mouse models of mitochondrial dysfunction, which might be mediated by attenuation of ISR [26]. DELE1 also has additional proapoptotic activities [32]. Thus, we speculated that activation of OMA1 and ISR could be pathogenic in optic nerve injury-induced RGC loss. In the present study, levels of ISR core mediators (p-eIF2α and nuclear ATF4) were increased after nerve injury. Both the PKR inhibitor C16 and the ISR inhibitor ISRIB were sufficient in promoting RGC survival. Zn^2+^ chelation reversed the upregulation of p-eIF2α expression and nuclear ATF4 distribution. In addition, combining Zn^2+^ elimination and ISR inhibition had no additive neuroprotective effects, suggesting that Zn^2+^-OMA1 and ISR potentially act in the same pathway in RGC fate determination. These results suggest a previously unknown pathogenic role of ISR in RGC death and provide a potential downstream molecular mechanism for the activation of Zn^2+^-OMA1 in optic nerve injury.

Further investigations are required to elucidate the effects of OMA1 knockout in RGCs in neuroprotection after optic nerve injury. In addition, whether OMA1 in ACs also regulates presynaptic Zn^2+^ release and postsynaptic RGC survival could be clarified in future studies. Besides the pharmaceutic inhibition of ISR, the effects of ATF4 or PKR knockdown in RGC protection after optic nerve injury are worth being considered in our future studies. To validate the potential retina–brain connection and visual function recovery, whether OMA1 knockout and ISR antagonists also regulate RGC axonal regeneration after optic nerve injury is worth investigating.

**Figure 9:**
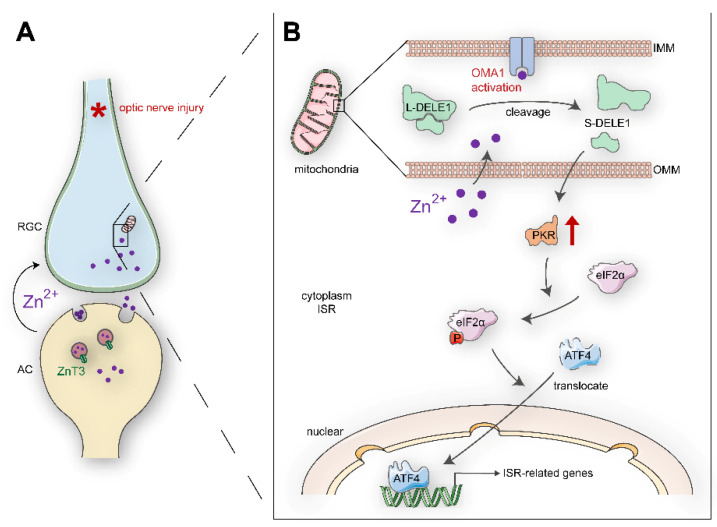
Schematic summary of results and potential signaling pathways: (**A**). Retinal AC-RGCs transsynaptic process of Zn^2+^ after optic nerve injury. (**B**). In postsynaptic RGC soma, Zn^2+^ induces mitochondrial structural damage and activates mitochondrial zinc metallopeptidase OMA1. The Zn^2+^-OMA1 mitochondrial signal increases DELE1 cleavage and PKR expression, which relays to a cytoplasmic ISR (eIF2α phosphorylation and ATF4 translocation). ISR-induced gene expression drives signaling toward RGC death. OMM, outer mitochondrial membrane; IMM, inner mitochondrial membrane.

In summary, our current study provided novel evidence demonstrating that RGC loss in optic nerve injury requires an increase in synaptic Zn^2+^ transfer that activates the mitochondrial zinc metalloprotease OMA1, resulting in postsynaptic mitochondrial crista disruption and ISR due to enhanced DELE1 processing and PKR expression (Figure 9). Significantly, this cascade of events can be arrested at several stages: TPEN- or ZnT-3 deletion induced reduction in Zn^2+^-OMA1 crosstalk, C16-mediated PKR inhibition, or ISRIB-mediated ISR inhibition (Figure 8E). All of these interventions result in the preservation of RGCs. Thus, OMA1 and the ISR are potential therapeutic targets for the prevention and treatment of neurodegenerative diseases.

## Data Availability

All data needed to evaluate the conclusions in the paper are present in the paper and/or the Supplementary Materials. Raw RNA-seq data were submitted to the Gene Expression Omnibus, National Center for Biotechnology Information (GEO-NCBI) (accession number: GSE208777).

## Supplementary Materials

The following supporting information can be downloaded at: https://www.mdpi.com/article/10.3390/antiox11102001/s1, Figure S1: Zn^2+^ induced mitochondrial disruption and OMA1 activation; Figure S2: RGC post-sorting analysis and dose-response survival of RGCs to ISR inhibitors.
