# Supplementary material for: Increased Mobile Zinc Regulates Retinal Ganglion Cell Survival via Activating Mitochondrial OMA1 and Integrated Stress Response
Source: Antioxidants (Basel). 2022 Oct 10;11(10):2001. doi: 10.3390/antiox11102001 (PMC9598227; doi:10.3390/antiox11102001)
Supplement: Supplementary file 1 [file antioxidants-11-02001-s001.zip › antioxidants-1940678-SI.pdf]

# Increased mobile zinc regulates retinal ganglion cell survival via activating mitochondrial OMA1 and integrated stress response

**Jiahui Tang, Zhe Liu, Jiaxu Han, Jingfei Xue, Liyan Liu, Jicheng Lin, Caiqing Wu, Qi Zhang, Siting Wu, Canying Liu, Haishun Huang, Yuanyuan Fu, Min Li, Yehong Zhuo *, Yiqing Li ***

**Supplementary Materials:**

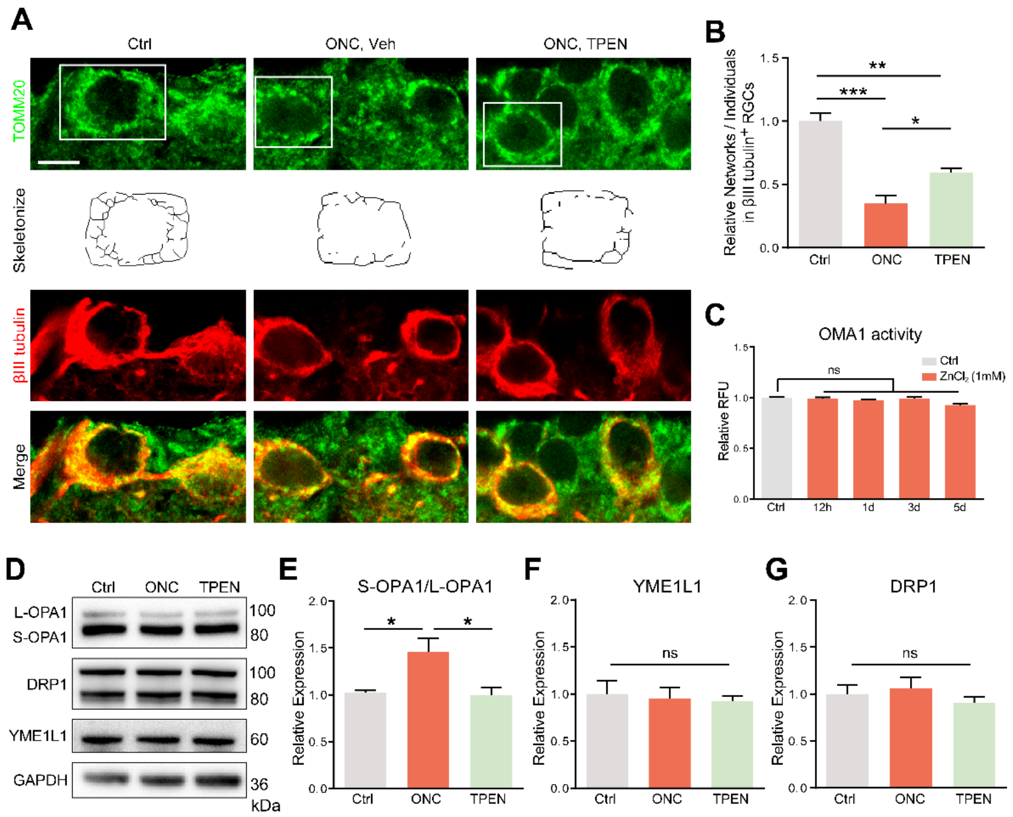


**Fig. S1. $Zn^{2+}$ induced mitochondrial disruption and OMA1 activation.**

(A). TOMM20 and βIII tubulin immunofluorescence staining in control and optic nerve injury murine retinas and the mitochondrial skeletonization. Scale bar, 10 μm. (B). Quantitation of mitochondrial network/individuals in βIII tubulin-positive RGCs. n = 3. One-way ANOVA with Bonferroni's post-hoc tests, *** $P < 0.001$, ** $P < 0.01$, * $P < 0.05$. (C). OMA1 activity in the retina before and after intraocular injection of 1 mM $ZnCl_2$, n = 4. One-way ANOVA with Bonferroni's post-hoc tests. Values are normalized to that of normal control retinas. ns, no significance. (D-G). Western blotting and quantification of OPA1 expression (n = 3), DRP1 (n = 5), and YME1L1 (n = 5) in the retina (GAPDH as a loading control, normalized to normal control). One-way ANOVA with Bonferroni's post-hoc tests, * $P < 0.05$; ns, no significance. All bars show the mean ± SEM.

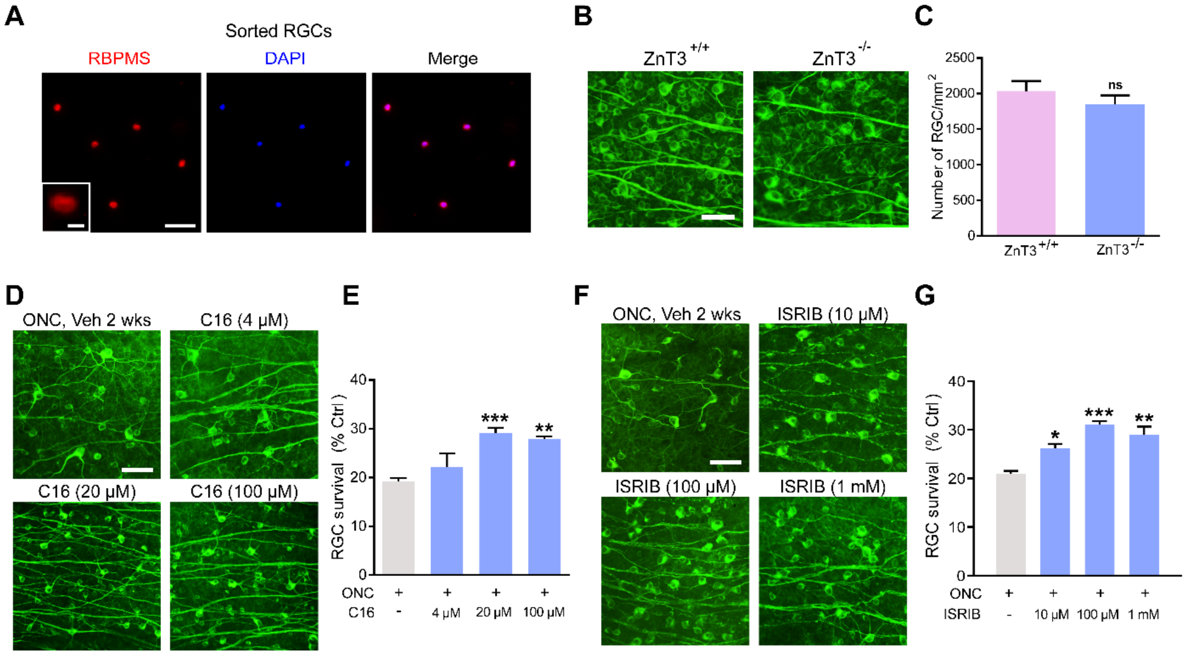


**Figure S2. RGC postsorting analysis and dose-response survival of RGCs to ISR inhibitors.**

(A). Representative images of sorted cells by flow-cytometry showing the intracellular localization of RBPMS, an RGC specific intracellular marker, overlapping with DAPI nuclear staining. Scale bar, 50 μm (lower magnification), 10 μm (higher magnification). (B, C). RGC counts in retinal flat mounts from uncrushed ZnT-3$^{-/-}$ and ZnT-3$^{+/+}$ littermates, n = 3. Unpaired t-test; ns, no significance. Scale bar, 50 μm. (D, F). Retinal flat mounts immunostained for βIII tubulin to visualize RGCs in normal control mice or 2-wk post-ONC, with treatments as indicated. Scale bar, 50 μm. (E, G). Quantitative results. n > 4. One-way ANOVA, Bonferroni's post-hoc tests. *** P < 0.001, ** P < 0.01, * P < 0.05, compared with vehicle-treated controls. All bars show the mean ± SEM.
